# Supplementary material for: Are carbon emissions trading and green financial instruments synergistic? -Comprehensive quantitative research based on content analysis
Source: PLoS One. 2024 Mar 7;19(3):e0298601. doi: 10.1371/journal.pone.0298601 (PMC10919601; doi:10.1371/journal.pone.0298601)
Supplement: S1 Data — (ZIP) [file pone.0298601.s001.zip › Data/地级市Z_F工作报告.zip!/╔╜╢1⁄2/┴┘╥╩╩╨/└┤╫╘╣1⁄2╓┌║┼í╛┬φ┐╦╩2╛▌═°í┐_2014.docx]

各位代表：

现在，我代表市人民政府向大会报告工作，请予审议，并请市政协委员和其他列席会议的同志提出意见。

一、2013年工作回顾

过去一年，在市委坚强领导和市人大、市政协及社会各界监督支持下，市政府团结带领全市人民，深入贯彻科学发展观，坚决执行中央和省委、省政府决策部署，创造性地落实“四三二一”总体思路，着力推进科学跨越发展，圆满完成了年度目标任务。

——经济发展稳中向好。全市实现生产总值3336.8亿元，增长11%；公共财政收入216.1亿元，增长16.6%，税收占财政收入比重达到85.3%；三次产业比例调整为9.7:47.5:42.8，服务业增加值占比提高1个百分点。投资、消费、出口协调拉动，固定资产投资、社会消费品零售总额、进出口总额分别增长20.8%、13.4%和19.1%。县域经济“三年倍增计划”如期完成，11个县区、开发区公共财政收入过10亿元，其中1个过50亿元，9个县在全省县域收入排名中实现位次前移。

——生态建设增添亮点。成功创建国家森林城市，新增国家级湿地公园6处。被列为全国水生态文明建设试点城市，沂河成为国家级水产种质资源保护区。累计创建国家级生态示范区7个，有6个镇(村)入选全国美丽乡村创建试点。节能减排力度加大，全市万元GDP能耗完成省政府下达任务，42个重点河流断面水质综合达标率为93.8%。

——文化软实力持续提升。习近平总书记视察临沂时指出，沂蒙精神与延安精神、井冈山精神、西柏坡精神一脉相承，是党和国家的宝贵精神财富。“十二五”广播电视“村村通”任务提前完成，市群众艺术馆、图书馆被评为国家一级馆，现代柳琴戏《沂蒙情》等作品获国家级大奖，被评为文化强省建设先进市。十二届全运会上获7枚金牌，创历史最好成绩；成功举办第二届中国沂河国际运动娱乐节，被国际滑水联合会授予“世界滑水之城”称号。

——群众生活进一步改善。各级财政民生支出263.3亿元，占公共财政总支出的64.9%。城镇居民人均可支配收入27511元，增长12.5%；农民人均纯收入突破万元、达到10389元，增长13.6%，增幅连续4年高于城镇。在中央电视台经济生活大调查中，临沂跻身居民幸福感最强的十大地级城市。

——城市影响力明显增强。通过国家知识产权试点城市验收，被认定为全国科技进步先进市；连续四年入选福布斯“中国大陆最佳商业城市”50强；在全国城市文明指数和未成年人思想道德建设两项工作测评中，得分均列地级城市第一名；沂蒙党的群众路线教育基地被国家行政学院、浦东干部学院、中直机关党校等院校列为现场教学点，全年接待各类培训班386个、2.8万人次。临沂得到各级领导和客商的好评，知名度和美誉度日益提高。

一年来，我们主要做了以下工作：

(一)突出产业强市，加快打造经济升级版。把构建现代产业体系作为经济工作总抓手，科学谋划、全力实施“10+6”产业推进计划、“1531”骨干企业培植计划和“1332”国际商贸名城建设计划，开启了产业转型发展新征程。强化产业支撑。“10+6”产业产值、200家骨干企业主营业务收入分别占规模以上工业的92.7%和33.6%，主营业务收入过百亿元企业达到10家、新增2家；临沂商城年交易额达到2096.2亿元，直接出口额增长150.8%；亿元以上新开工项目突破1000个，完成投资过亿元项目799个，分别增加677个和403个。优化产业结构。农业基础地位更加稳固，粮食等主要农产品供求平衡，新发展优质农产品园区138个，新认证“三品一标”128宗，新建水利工程2205项、高标准农田52万亩。工业升级步伐加快，总产值突破万亿元，产值过亿元、利税过千万元企业分别新增423家和405家，被认定为国家工程机械高新技术产业化基地。服务业质效提升，24个现代服务业集聚园区入驻企业1300多家，蒙山旅游区成功创建国家5A级旅游景区，物流业增加值占GDP比重保持全省首位。

(二)突出城乡统筹，加快一体化发展。把新型城镇化作为发展的最大潜力，着力构建中心城区、县城、小城镇和农村新型社区“四级联动”的现代城镇体系，城镇化率达到53.2%。提升中心城区带动力。建成区面积、人口分别达到195平方公里、193万人；江泉高架路、沂河路-西外环立交桥、临工大桥、30万吨东线供水等市政重点工程进展顺利。增强县城和小城镇承载力。县城城建投资和村镇建设投资分别完成20亿元、110亿元，12个镇被列为第二批省级示范镇。推进农村新型社区建设。新建农房9.5万户、危房改造2万户，实施“两区同建”的社区114个。完善立体交通体系。干线公路和农村公路分别新改建176公里、2158公里，青临高速建成通车，实现高速公路“县县通”和村级公路网化示范工程县域全覆盖；新开通航线4条，每周航班达107个。加强城镇规划和管理。新一轮中心城区控制性详规和城市设计覆盖面扩大，县城总体规划和省级示范村庄规划编制基本完成。行政村规模布局得到优化，数量由7151个减少到3990个。城乡环境综合整治深入开展，污水日处理能力达95万吨，在全国地级市中率先实现天然气“镇镇通”。

(三)突出改革创新，加快增强发展活力。把破解瓶颈制约作为科学跨越发展的突破口，扎实推动重点领域和关键环节的改革创新。推进节约集约用地。在全省率先完成新一轮地价更新，验收增减挂钩指标2万亩，有效利用批而未供土地6.6万亩，土地市场动态监管走在全国前列，集体土地所有权确权登记发证顺利完成。完善金融服务体系。新引进股份制商业银行5家，新兴金融业态注册资本过百亿元，金融机构本外币存贷款余额分别为3710.8亿元和2531.1亿元，存贷比达到68.2%，社会融资规模增长34.7%。加快科技创新。新增博士后科研工作站6家、院士工作站16家，55%的规模以上工业企业开展产学研合作，临沂技师学院成为国家级高技能人才培训基地。改善营商环境。市级行政审批事项由216项减少到110项,成为全省审批事项最少的市之一；“12345”市民服务热线试运行，受理群众诉求33万件；放宽市场准入，新发展市场主体4.7万户，1.2万家个体工商户升级为企业。

(四)突出民生优先，加快建设幸福美好家园。把人民群众对美好生活的向往作为奋斗目标，集中力量办好民生十件大事和二十项实事。新增城镇就业13.2万人、农村劳动力转移就业23.2万人。新型农村和城镇居民社会养老保险并轨，企业退休人员基本养老金人均月增248.4元。城乡低保对象应保尽保，新建保障性住房22648套，新建、扩建敬老院52处。中小学校舍安全工程五年规划全面完成，新建、改扩建幼儿园388所，临沂大学“自然出版指数”列全国高校第八位。新农合参保率、大病补偿金额居全省首位，国医堂建设和传染病防控保持全省领先。计生工作有效开展，低生育水平保持稳定。社会治安防控体系进一步完善，信访秩序持续好转，食品药品监管有力，安全生产形势平稳。

(五)突出为民务实清廉，加快提升政府工作水平。坚持依法科学民主决策，凡“三重一大”事项均集体研究决定，涉及群众利益的重要决策都广泛征求群众意见。自觉接受人大法律监督、政协民主监督和群众社会监督，人大代表建议和政协提案均按时办理。扎实开展政策措施落实年、服务企业“四比四看”活动，严厉整治“庸懒散”行为，工作集中度和执行力明显提高。认真贯彻中央“八项规定”和国务院“约法三章”，“三公经费”支出下降7.1%。严格执行党风廉政建设责任制，有力促进了干部清正、政府清廉、政治清明。

各位代表，2013年全市各条战线都开拓创新，奋发有为。国防动员、人民防空、民兵预备役建设、双拥共建工作不断加强，妇女儿童、慈善、残疾人、老龄事业持续发展，民族、宗教、对台、外事、侨务、贸促会、统计、史志、档案、保密、盐务、应急管理、气象和防震减灾等各项工作都取得新的进步。

去年成绩来之不易。这是省委、省政府和市委正确领导的结果，是历届市政府接力奋斗的结果，是全市人民团结拼搏的结果，也是各方面关心帮助的结果。在此，我代表市人民政府，向全市干部群众，向人大代表、政协委员、各民主党派、工商联、人民团体和离退休干部，向驻临人民解放军、武警官兵和中央、省属单位，向在我市投资兴业的各地客商和外来务工人员，向支持临沂发展的各界朋友，表示崇高敬意和真诚感谢!

各位代表，一年来的实践启示我们：发展离不开战略。把握发展的主要问题，才能达到“牵一发而动全身”的突破；抓住矛盾的主要方面，才能取得“一子落而满盘活”的效果。推动跨越赶超，必须把临沂放到全省、全国的大格局中审视，把握发展规律，顺应阶段特征，科学顶层设计，确立战略目标、路径选择和工作重点，让发展有方向、有遵循。科学发展需要科学思维。临沂作为相对欠发达城市，实现后发崛起，必须用系统思维谋划发展，用辩证思维认识问题，用开放思维借力发展，用底线思维应对挑战，靠科学的方法论指导实践，努力走出有区域特色的科学发展之路。转型发展势在必行。提高经济质量效益，实现不会带来后遗症的发展，关键在于转变经济发展方式。必须坚持腾笼换鸟、凤凰涅槃的思路，认真落实市委关于“五个更加注重”的要求，加快实现工作指导上的重大转变，优化产业布局，搞好要素组合，将优质资源配置给最能发挥效用的市场主体，坚决打好转方式调结构攻坚战。改革创新是最大红利。解放和发展生产力，必须充分看到改革蕴藏的巨大潜力、体制机制变革包含的强劲动力，顺应改革大势，凝聚改革共识，着力实施思路创新、模式创新、机制创新和科技创新，以创新破解瓶颈、聚集要素，推动发展。群众满意是政府的第一追求。让老百姓过上好日子是我们一切工作的出发点和落脚点。实现富民强市目标，必须以更大力度保障和改善民生，及时有效回应社会诉求，解决好群众最关心最直接最现实的利益问题，在增进人民福祉中集聚发展正能量，在为群众排忧解难中提高社会和谐度。打铁还需自身硬。赢得人民的信任和支持，必须提高驾驭市场经济和应对复杂局面的能力，增强政府公信力、干部执行力和制度约束力，切实做到公权为民、廉洁从政、勤勉敬业、创先争优，树立团结实干、开拓奋进的政府形象。

在看到成绩的同时，我们也清醒认识到：市场有效需求不旺、经济面临下行压力，实现“与全省同步全面建成小康社会”目标任务艰巨；传统产业多数处在价值链低端，新兴产业占比较小，资源环境约束趋紧，“转调创”力度亟待加大；城乡基础设施建设和社会事业发展相对滞后，有些涉及群众切身利益的问题需要进一步解决；机关作风与广大人民群众的期盼仍有差距，营商环境有待持续优化。我们必须强化问题导向，研究针对性措施，加倍努力工作，不断创造无愧于使命和时代的业绩!

二、2014年工作安排

今年是完成“十二五”规划任务的关键一年。综观国内外形势，我们既面临挑战，也有许多机遇。习近平总书记来临沂视察，为我们指明了努力方向；我市参照执行国家中部地区有关政策，列入山东西部经济隆起带规划，成为省级国际贸易综合改革试点，多项利好政策迭加；民营经济活跃，商贸物流发达，资源优势突出，发展基础坚实。只要我们准确把握上级精神，坚定信心、顺势而为、精准发力，就一定能够开创经济社会发展新局面。

今年政府工作总的要求是：深入贯彻党的十八大、十八届三中全会和习近平总书记系列讲话精神，坚持以科学发展观为指导，以稳中求进、进中求好、好中求快为基调，以提高经济发展质量和效益为中心，以改革创新为动力，加快实现工作指导重大转变，认真落实“四三二一”总体发展思路，着力构建现代产业、现代城镇和民生保障“三个体系”，实施“10+6”产业推进、“1531”骨干企业培植和“1332”国际商贸名城建设“三项计划”，建立健全营商环境、要素供应和责任落实“三个机制”，努力保持经济持续健康发展和社会和谐稳定。

主要预期目标是：生产总值增长11%左右，公共财政收入增长14%以上，固定资产投资增长20%以上，社会消费品零售总额增长13%以上，进出口总额增长14%以上，城镇居民人均可支配收入、农民人均纯收入分别增长11%和12%，居民消费价格涨幅控制在3%左右，城镇登记失业率控制在3.5%以内，人口自然增长率、万元GDP能耗和主要污染物排放总量削减率等约束性指标，确保完成省政府下达的任务。

在复杂经济环境条件下，实现科学跨越发展，必须把握好事关全局的三个关键问题。第一，把握可持续发展问题。能耗高、污染重、附加值低、产业链条短等问题，已经严重制约了我市经济竞争力的提升。这要求我们下决心摆脱对资源消耗和环境投入的过度依赖，坚持做到“招商不忘选商、落地不忘节地、生产不忘生态、制造不忘创造、富市不忘富民”，努力实现资源节约和环境友好目标。第二，把握质量效益导向问题。就是把以质量效益为中心贯穿于经济发展各方面、推动工作全过程、谋划发展各环节。无论是构建现代产业体系、培植骨干企业、建设国际商贸名城，还是推进新型城镇化；无论是项目实施，还是园区建设，都要坚持速度与质量效益相统一。第三，把握转方式调结构问题。转变经济发展方式，就要转变发展的方法、手段和模式。当务之急是研究好“转什么、怎么转、转得怎么样”，“调什么、怎么调、调得怎么样”，“创什么、怎么创、创得怎么样”等问题。特别要用心思考产业发展方向，明确鼓励发展什么、限制和淘汰什么；如何加快农业链条向两端延伸，前端发展生产资料和农业装备制造，后端发展精深加工和品牌营销，让农业成为有奔头的产业；如何化解工业落后产能，加快将众多低端的冶金、规模庞大的木业、高耗能链条短的化工、低效污染重的陶瓷等传统产业，进行有计划地改造提升，形成持久竞争力；如何加快发展战略性新兴产业，在新能源、信息技术、先进装备制造、节能环保、新材料等重点领域实现突破，打造新的优势产业；如何优化提升旅游、商贸等传统服务业，发展金融保险、总部经济、服务外包等现代服务业，推行电子商务等新型业态，加快打造服务业区域性中心；如何搭建创新平台，促进科技成果转化，提高全要素生产率，努力以“转调创”的主动，赢得区域发展和竞争的主动。

具体工作中，重点抓好六个方面：

(一)着力构建现代产业体系，加速转型升级。全力落实“三项计划”，狠下功夫，痛下决心，坚决化解落后产能，腾出发展空间，集中要素资源，抓好存量调整和增量优化，努力提高第三产业比重和最终产品、绿色环保产品、有记录可追溯产品的比重。

发展壮大现代农业。突出做到三个确保、四个提高：三个确保，即确保粮食安全，圆满完成粮油储备任务；确保全市农产品质量安全抽检合格率稳定在98%以上，争创全国农产品质量安全放心市；确保农民人均纯收入增幅高于GDP，各项惠民政策得到有效落实。四个提高，即提高规模化经营水平，依法推进农村土地经营权流转，积极培育新型经营主体，新发展家庭农场200家，新增工商注册农民专业合作社1000家；提高标准化生产水平，抓好“三条生态长廊、四大板块”建设，新建优质农产品产业园区50个；提高品牌化营销水平，新培育影响力强的区域公共品牌10个，进一步打响“生态沂蒙山、优质农产品”品牌；提高装备现代化水平，农机化作业综合水平达到85%，改善和恢复有效灌溉面积62万亩，新建高标准农田75万亩，再解决50万农村人口饮水安全问题。

调优做强工业。突出做到四个坚定不移。一是坚定不移地把“10+6”产业作为转型发展的主战场。发挥16个推进办、15个发展主体、7个涉外招商部门三支队伍作用，实施补链、强链、建链三项工程，打好招大引强和项目建设提速“两个百日战役”。深化“三拜访一下访”，对接央企、500强、行业龙头、科研机构，大力引进龙头型、旗舰型、基地型项目，力争签约项目实际到位资金900亿元。健全完善项目前期落地、中期挂牌督办、后期服务保障机制，力争“10+6”产业完成固定资产投资2400亿元，增长26%左右；实现产值或收入12600亿元，增长25%左右。二是坚定不移地把“1531”骨干企业作为转型发展的主力军。着重实施提升企业战略、完善法人治理结构、改造工艺装备、突破科技研发、创新营销方式“五大工程”，支持企业抓好技术改造和市场化兼并重组，增强核心竞争力，争取工业技改投资增长22%以上，200家骨干企业主营业务收入、利税分别增长17%、16.5%。同时，实施千企培育计划，今后三年每年重点扶持300家以上潜力型中小企业，不断壮大骨干企业群体。三是坚定不移地把新兴产业作为转型发展的新亮点。鼓励企业加强产业链高端产品研发，推进信息化和工业化深度融合，力争在节能环保、物联网、云计算、大数据等重点领域实现突破，形成新的产业优势。四是坚定不移地把各类开发区作为转型发展的大平台。以两个国家级开发区为龙头，以临港等省级开发区为主体，推行“一区多园”模式，实行“飞地经济”政策，引导产业向优势区域集聚，构筑有市场话语权的产业高地。

优化升级服务业。整体提升商贸物流、旅游、金融等主导型产业，着力培育软件和信息服务、文化创意、商务服务、研发设计等潜力型产业，积极发展教育培训、养老服务、医疗健康服务等紧缺型产业，加快服务业做强优势、补齐短板、提质增效。突出发展服务外包与总部经济，加大对生产性服务业的扶持力度，确保中印国际软件园建成运营，东忠科技园等项目顺利推进。抓好中国兵学城等文化产业项目建设，扶持发展影视制作、动漫设计等创意产业，争创国家版权示范城市。加快第四代移动通信技术商用化进程，有序推进“三网融合”。围绕建设旅游目的地城市，策划引进大型体验式、龙头型项目，实施蒙山改造提升计划，开展乡村旅游示范乡镇创建活动，集中推介精品线路，争取接待游客突破5000万人次。

促进县域经济崛起。启动新一轮县域经济发展计划，打造中心城区核心经济板块、东部临港经济板块、南部临郯苍经济板块和西北部高效生态经济板块。鼓励县区按照“一个主导产业、一批龙头企业、一批配套企业、一个区域品牌”模式，极化优势、差异竞争，大力发展特色经济和产业集群，加快增强经济实力，力争全市公共财政收入过20亿元的县区、开发区达到4个。以22个省级示范镇和12个市级优先发展镇为重点，引导和支持重点镇优先发展、中心镇加快发展、其他乡镇竞相发展，建设一批工业主导型、商贸带动型、现代农业型和旅游休闲型小城镇。

(二)着力深化改革，推动创新发展。正确、准确、有序、协调推进改革，重点处理好政府和市场的关系，争取在产业转型、金融创新、文化等领域求突破。

深化经济体制改革。扎实搞好财税改革，稳步推进“营改增”试点和地方税体制调整；建立“全口径”政府预算体系，扩大预算绩效管理范围，形成“花钱必问效、无效必问责”机制；加大政府购买服务力度，鼓励社会力量参与公共服务。创新金融服务，强化金融监管，推动由民间资本发起设立银行，深化农村信用社改革，规范发展民间融资，建设齐鲁股权交易中心临沂管理中心和市总商会中小微企业金融服务中心，完善现代金融体系，加快打造区域金融中心，力争社会融资规模增长20%以上。加快国企改革步伐，完成新华印刷物流集团等6家国企改制。扶持发展非公有制经济，支持非公有资本参与国有企业改制重组和合资经营；发挥工商联服务非公经济的作用，大力培育发展各类行业商会和异地商会，培养造就高素质企业家队伍。完善“监、管、办”分离的公共资源交易管理体制和运行机制，坚决杜绝场外交易。扎实推进农村产权制度改革，建立农村产权流转交易市场，开展农村产权融资试点，激活农村生产要素。

深化行政管理体制改革。进一步简政放权，最大限度减少政府对微观事务的干预。健全四级服务网络，建设集便民服务、行政审批、公共资源交易等于一体的市行政服务中心。依法调整涉企收费项目和标准，推动中介机构服务“去行政化”，努力降低企业和群众创业成本。加快工商注册制度便利化改革，及时实行“负面清单”、“先照后证”、“企业年度报告公示”制度。出台优化营商环境的若干规定，坚持“事不过一”原则，严厉查处违规行为，加快打造全省审批事项最少、收费标准最低、办事效率最高、营商环境最优的城市之一。

深化文化体制改革。加快国有文化企业公司制、股份制改造，实施小微文化企业孵化培养计划，发展文化中介机构，引导社会资本参与对外出版、网络出版；健全文艺创作激励机制，深化公益性文化事业单位内部机制改革，扶持重点文化企业，加快文化资源和产品开发，打造一批体现沂蒙特色的文化品牌。发挥沂蒙革命纪念馆的作用，大力弘扬奋发向上、崇德向善的社会风尚，争创全省“四德工程”建设示范市。

完善创新驱动发展机制。强化企业在技术创新中的主体地位，加快骨干企业创新平台全覆盖，建立“临沂产学研协同创新联盟”和“人才创新创业基地”，新建院士工作站10个，引进高层次人才100名。抓好临沂应用科学城等公共创新载体建设和管理，推进国家新能源汽车应用推广示范城市建设，争创国家火炬计划新能源电动车特色产业基地和山东省“物流科技创新示范基地”。建立重大科技项目协调推进机制，组织科技创新项目攻关，集中力量破解制约发展的关键核心技术。深入实施质量强市和品牌带动战略，支持企业创造新品牌、制定新标准，实现管理创新、商业模式创新，争取新创驰名商标、著名商标和省级以上名牌产品50个以上。加快科技成果转化，争取高新技术产业产值占规模以上工业总产值比重提高1.5个百分点。

(三)着力构建现代城镇体系，做优发展载体。坚持以人的城镇化为核心，以保发展、保民生、保安全为导向，以建好城、繁荣市、满足人为目标，不断提高规划建设管理水平，力争列为全省新型城镇化试点城市，城镇化率达到55%左右。

高起点、全市域规划。强化“三规合一”、“四级联动”，合理确定不同功能区的发展定位和规模布局，优化城镇空间形态。科学编制中心城区公共服务设施布局等专项规划，搞好县城重点区域、重要地段的控规和专项规划，加大县城规划编研力度，提高控规覆盖率，推进乡镇新一轮总体规划修编，完善提升第二批省级示范镇规划，加大村庄(社区)规划编制力度，加快形成高水平、有特色、有权威的城镇规划体系。对城区拟开发地块和建设项目，一律先行搞好规划编研，严格按照确定的容积率、基本形态、基本地价和设施配套等要求，进行招商建设，切实维护城市发展的整体性、统一性。

高质量、大力度建设。加大基础设施投入。组织实施“四供两排”等十大工程和一批重大交通设施项目，做好鲁南铁路(高铁)客运专线临沂—曲阜段建设的前期工作，开工建设临沂铁路物流园、临沂火车站改造提升工程，确保沂沭铁路、东外环等建成，西安路祊河桥和南京路、陶然路沂河桥等工程顺利实施，“三环十五射”现代城市道路体系基本形成；启动中心城区“双千万”热源建设计划，今明两年每年新增供热能力1000万平方米；加快30万吨供水工程开发，力争日供水18.6万吨；扩大“气化临沂”成果，中心城区新增天然气用户8万户；加快与国家电网合作，力争沂蒙抽水蓄能项目开工建设。积极推进产城融合。在中心城区合理布局高端商务圈，加快城市综合体建设，大力发展城市经济；在县城和中心镇重点发展特色二、三产业和现代农业，促进农村人口就近转移；深入实施农村社区和产业园区“两区同建”，培育不少于3000人具备城镇功能的农村新型社区70个，并将符合标准的纳入城镇化管理。

高效能、规范化管理。按照“两级政府、三级管理、四级网络”要求，构建“无缝隙、全覆盖、全天候”城市管理体系。整合地理信息、数字城管、社会治安等公共服务信息资源，提高数字化指挥调度平台建设水平。加强渣土运输、露天烧烤、违法建设、户外广告和市容环境整治，推动环卫保洁城乡一体化。推广“大城管”经验，建立齐抓共管的长效机制。

(四)着力推进商城国际化，扩大对外开放。坚持以内外贸一体化发展带动产业集聚，以商城国际化推动产业提档升级，争取列为国家级国际贸易综合改革试点，商城年交易额超过2500亿元、直接出口额超过60亿美元。

加快国际商贸名城建设。提升载体功能，在遵循和延续原有城市空间布局的基础上，以祊河为轴，在北城新区新建国际商务区和现代流通综合示范区，建设电子商务大厦、现代物流大厦和国际采购大厦等重点项目；在老城区实施“五个一批”计划，促进现有市场提升改造，加快经营主体、交易方式、流通体系和保障手段的现代化。发展新兴业态，启动电子商务“十百万”工程，打造集第三方支付、国际结算、大数据中心等功能于一体的“电商金融大平台”，构建实体和虚拟经济相结合的商业新模式，全市电子商务交易额实现600亿元；完善现代物流体系，优化节点布局，支持发展第三方物流，推广甩挂运输、集装箱多式联运等先进方式，力争物流业增加值达450亿元；举办好第五届商博会、首届中国·临沂资本交易大会，力争实现意向融资1000亿元以上，会展业展出面积75万平方米。

更加有效地开展对外经济合作。加快构建国际贸易采购体系，成立国际贸易采购体系服务中心，用好中国(临沂)跨国采购中心这一政府主导公共服务平台，为外贸企业提供综合公共服务；强化涉外服务，搞好综合保税区筹建和航空口岸开放争取工作；培育参与市场采购的本市外贸公司100家，引进外贸500强等国内涉外经营主体100家、境外采购商2000名以上；主动对接口岸城市，设立境外招商联络处，全面拓展国际化联系网络。千方百计引进和利用外资，组织参加香港山东周、鲁台会等重大招商活动，支持符合条件的企业到境外上市融资，鼓励外资以参股、并购等方式参与我市企业的改组改造，广泛吸引私募股权投资。审慎推进企业对外投资，加快中欧商贸物流合作园区建设，支持优势产业企业并购境外研发中心和知名品牌，建设境外资源合作开发基地。

(五)着力加强生态临沂建设，促进可持续发展。坚持生态优先，大力发展绿色、循环、低碳经济，争取第一批列入国家“生态文明先行示范区”。

强化资源节约集约利用。严守耕地红线，加大增减挂钩、有效供地、执法监管、集约用地四项重点工作力度，鼓励新开工项目建设多层标准厂房，搞好工矿废弃地复垦利用、低丘缓坡等未利用土地开发，争创全国国土资源节约集约模范市。全面执行水资源开发利用“三条红线”，深入推进全国水生态文明城市试点工作，积极创建国家节水型城市。加快矿业结构调整，建设“绿色矿山”，提高集约化开发水平。抓好“两型企业”创建，加强工业固体废物利用，新增清洁生产审核单位100家以上。

强化节能减排。认真执行节能评估审查制度，实施差别电价和惩罚性电价政策，严控“两高一资”和落后产能项目上马；加快居住和公共建筑节能改造，创建国家可再生能源建筑应用示范城市。完善水质监测监管和预警应急体系，千方百计减少污染物排放，确保饮用水水源地水质达标率100%；健全“治、用、保”流域污染防治体系，抓好底泥生态修复、养殖业污染治理、入河直排口整治和面源污染防治，大幅提高断面水质保障水平，坚决打赢迎淮考核战役。

强化大气污染治理。落实大气污染防治行动计划，下大气力治理雾霾。推进钢铁、焦化、建陶、水泥等行业结构调整，加快重点行业治污提升，坚决取缔关停中心城区页岩砖厂、石灰窑等污染源；加强建筑施工、道路运输、铁路货场、粉料储运、混凝土搅拌和城乡结合部扬尘污染综合治理；集中整治燃煤锅炉，大力发展清洁能源；强化机动车环保检测，严格防控机动车尾气污染；抓好秸秆禁烧，加强秸秆综合利用、离田收储等措施，切实改善大气环境质量。

强化绿色建设。启动生态补偿机制试验区建设，实行最严格的源头保护、损害赔偿和责任追究制度，构筑沂蒙山区绿色屏障。实施中心城区广场公园建设、生态片林和经济林营造等“五个一”工程，搞好“八河”治理及景观提升，加快形成沿河慢行绿道和“碧水绕城”的水系景观。加大城乡环境综合整治力度，继续抓好生态文明乡村建设，创建沂蒙“美丽乡村”100个。实施三年造林绿化工程，年内新造林26万亩，提升60万亩环城林带建设水平，力争森林覆盖率达到34.5%。

(六)着力完善民生保障体系，增进群众福祉。不折不扣地落实惠民政策，优先保障民生投入，着力办好教育惠民、养老助残等“十件为民实事”，加快构建幸福和谐家园。

保障底线民生。实施贫困劳动力培训“雨露计划”，扶持160个省级、40个市级贫困村搞好整村扶贫开发；有序推进公租房和廉租房并轨，探索出台农民工等外来务工人员的住房保障政策，开工建设公租房5000套、城市棚户区改造2.1万套；进一步提高社会救助标准，保障困难群众的基本生活；出台重特大疾病医疗救助办法，提高低保、五保对象政策范围内自付住院费用的医疗救助比例；用好价格调节基金，稳物价、保民生；建立困境儿童生活保障制度，落实城镇“三无”老年人福利供养、重度残疾人和困难失能老年人护理补贴制度，进一步让困难群体感受到党和政府的温暖。

搞好基本民生。建立经济发展和扩大就业联动机制，突出抓好以高校毕业生为重点的青年就业，统筹做好农村转移劳动力、城镇困难人员、退役军人就业工作，新增城镇就业8.5万人、农村劳动力转移就业15.5万人。健全医疗、失业、工伤、生育保险市级统筹机制，完善社会保险关系转移接续政策，扩大异地就医结算平台建设。大力推进中小学办学条件标准化建设，确保到2015年通过国家对县域义务教育均衡发展的评估验收；深化“名校进北城”工程，实施新一轮城区学校三年联建工程，确保临沂一中北校区秋季招生、市体校新校区开工建设。深化医疗卫生体制改革，鼓励引导社会资本举办医疗机构，引进高层次人才，培训基层人才，增强医疗服务能力。加强社会养老服务，加快市老年养护院、市老年公寓建设。完善“九纵九横”公交线网，新增、更新新能源公交车各100台，着力缓解交通拥堵问题。整顿物业市场秩序，提高星级规范化服务水平。推动全民健身，建成启用市民健身中心，新建农村健身工程1000个，举办高规格的大型水上运动节会。广泛开展群众性文化活动，健全城乡互动的文明创建长效机制，确保蝉联“全国文明城市”。

促进社会和谐。创新社会治理，对流动人口、特殊人群、非公有制经济组织和社会组织实行规范化、人性化管理，争创全国社会治理和服务创新试验区。根据上级部署，落实“单独两孩”政策，综合治理出生人口性别比，促进人口均衡发展。强化安全生产责任，加强隐患排查治理和安全预防控制，坚决遏制重特大事故发生。完善突发危机事件应急管理和防灾减灾体系，做好抗震设防、重点传染病防控和各类灾害预防工作。健全市县乡村四级监管网络，加强市县两级执法队伍建设，切实保障食品药品安全。开展好第三次全国经济普查，确保数据准确。建设“平安临沂”，加大社会治安基础建设投入，搞好矛盾纠纷排查化解，严厉打击各类违法犯罪，全力维护社会稳定。完善普法教育机制，增强全社会法治观念。

各位代表，2014年工作任务繁重，需要全市各条战线共同努力，奋力拼搏。我们要进一步做好国防动员、人民防空、双拥共建工作，增进军政军民团结；支持妇女儿童、工会、共青团、老龄等工作，发展广播电视、档案、史志、民族、宗教、外事、侨务、对台、红十字会等事业，推动经济社会协调发展。

三、关于政府自身建设

面对新形势、新任务，各级政府要牢记习近平总书记“把老区人民的生活搞得更红火、更幸福”的殷切嘱托，大力弘扬沂蒙精神，切实提高政府履职能力和素质，加快推进政府治理体系和治理能力现代化。

建设学习型政府。增强学习自觉性，营造善学习、勤思考、重研究的良好风气。增强学习针对性，善于从主流理论和前沿知识中把握规律，从实践和群众中汲取智慧，全面提高政治业务素质。增强学习实效性，注重用科学理论、先进经验谋划和推动发展，全面提高驾驭工作的水平。

建设创新型政府。坚持解放思想、与时俱进，用心研究新情况、探索新办法，加快突破旧的思维定势，不断以科学可行的顶层设计引领发展。坚持用改革的手段、市场的办法，加快破解瓶颈制约，努力让一切创造社会财富的源泉充分涌流。

建设为民型政府。扎实开展党的群众路线教育实践活动，聚焦“四风”，解决问题，推动改进作风制度化、常态化。健全领导干部带头深入基层调查研究制度，完善直接联系和服务群众机制。办好“12345”市民服务热线，保证群众诉求“事事有回音、件件有着落”，打造最具影响力的政务品牌。

建设法治型政府。健全公众参与、专家咨询和政府集体决定相结合的决策机制，凡属“三重一大”事项，坚决做到充分发扬民主，严格履行程序，确保决策的民主性、合法性和科学性。规范行政行为，推行权力清单制度，依法公开权力运行流程等信息，提高政府公信力。自觉接受监督，严格执行人大及其常委会的决议决定，积极支持人民政协履行职能，认真听取民主党派、工商联、无党派人士、人民团体和新型社会组织意见，广泛接受新闻舆论和社会公众监督，让权力在阳光下运行。

建设务实型政府。建立“横到边、纵到底”的责任体系和“闭环式”工作链，制定路线图、时间表，做到时间倒推、工期倒排，以钉钉子精神，一件一件抓落实、一项一项求突破。推行“定诺、亮诺、践诺、评诺”制度，深化“庸懒散”专项治理，令出必动、雷厉风行，提高政府工作绩效。强化分类指导，战略性任务倡导“二八律”，阶段性重点工程组织大会战，常规性工作抓平稳运行，力求以重点突破带动整体工作上水平。

建设廉洁型政府。深入贯彻中央“八项规定”，认真执行《党政机关厉行节约反对浪费条例》，强化公务接待、公务用车、办公用房等方面的规范管理，继续压缩“三公经费”支出，最大限度地降低行政成本。全面落实党风廉政建设责任制，对腐败现象零容忍，严肃查处各类违法违纪案件，坚决纠正损害群众利益的不正之风，以反腐倡廉实际成效取信于民。

各位代表!美好愿景令人鼓舞，群众期待催人奋进。让我们紧密团结在以习近平同志为总书记的党中央周围，在省委、省政府和市委坚强领导下，科学务实、改革创新，同心协力、扎实工作，为建设富裕美丽“大临沂、新临沂”而努力奋斗!
